# Percutaneous creation of de novo superior vena cava/right atrium conduit for cardiac resynchronization therapy upgrade

**DOI:** 10.1016/j.hrcr.2024.06.004

**Published:** 2024-06-13

**Authors:** Faizan Rathore, James Mannion, Jonathan Lyne, Kevin Walsh

**Affiliations:** ∗ Electrophysiology Department, Beacon Hospital, Dublin, Ireland; † Regional Hospital Mullingar, Mullingar, Westmeath, Ireland; ‡ School of Medicine, University College Dublin, Dublin, Ireland

**Keywords:** Pacing, Cardiomyopathy, Complex device challenges, CRT, Vascular complications, SVC obstruction, Percutaneous intervention

**Key Teaching Points**

• Pacing-induced cardiomyopathy and superior vena cava (SVC) stenosis and obstruction post device implant is a relatively common entity.
• Despite its prevalence, a consensus to approach the problem is lacking.
• Various options have been recommended, but no comparison exists.
• We demonstrate a novel approach to this problem.
• Device upgrade and implantation with SVC obstruction can be addressed through a de novo channel within the mediastinum.

## Introduction

Superior vena cava (SVC) stenosis and obstruction post transvenous cardiac device insertion has been widely documented.^1^, ^2^, ^3^ It becomes particularly challenging in the setting of lead revision or device upgrade.^4^ Management options include SVC venoplasty, thrombus debulking, and anticoagulation.^3^^,^^5^, ^6^, ^7^ In complex cases, however, lead extractions, surgical bypass with reconstruction of SVC,^8^ or alternative devices not requiring venous access^9^ may be considered. We present an interesting case of approaching SVC obstruction by percutaneous reconstruction of a de novo conduit to facilitate cardiac resynchronization therapy (CRT) upgrade.

## Case report

A 46-year-old man presented to our center for consideration of resynchronization therapy using either conduction system pacing or conventional CRT approach. His background history included symptomatic complete heart block, necessitating dual-chamber (DDD) pacemaker insertion in 2013, with subsequent development of pacing-induced cardiomyopathy, necessitating a CRT upgrade in 2017. Four years later, the CRT system had to be surgically extracted owing to infection, pocket breakdown, and erosion of the device. An epicardial DDD pacemaker was implanted concomitantly and a computed tomography venogram at the time demonstrated focal SVC stenosis. Other medical history included systemic lupus erythematosus, paroxysmal atrial fibrillation/atrial flutter, and normal coronary arteries.

On presentation the electrocardiogram was consistent with paced rhythm. Echocardiogram and cardiac magnetic resonance imaging confirmed left ventricular (LV) dysfunction (30%–35%). Device check demonstrated no underlying rhythm with consequent 100% ventricular pacing. Venogram demonstrated complete occlusion of SVC.

Based on the findings above, the options of surgical implantation of epicardial LV lead, implantation of LV electrode (WiSE CRT system; EBR Systems Inc, Sunnyvale, CA), and percutaneous intervention by creating a de novo conduit within the mediastinum, inspired by the innominate vein turn-down procedure,^10^ were offered to the patient, who opted to undergo percutaneous intervention.

**Figure 1:**
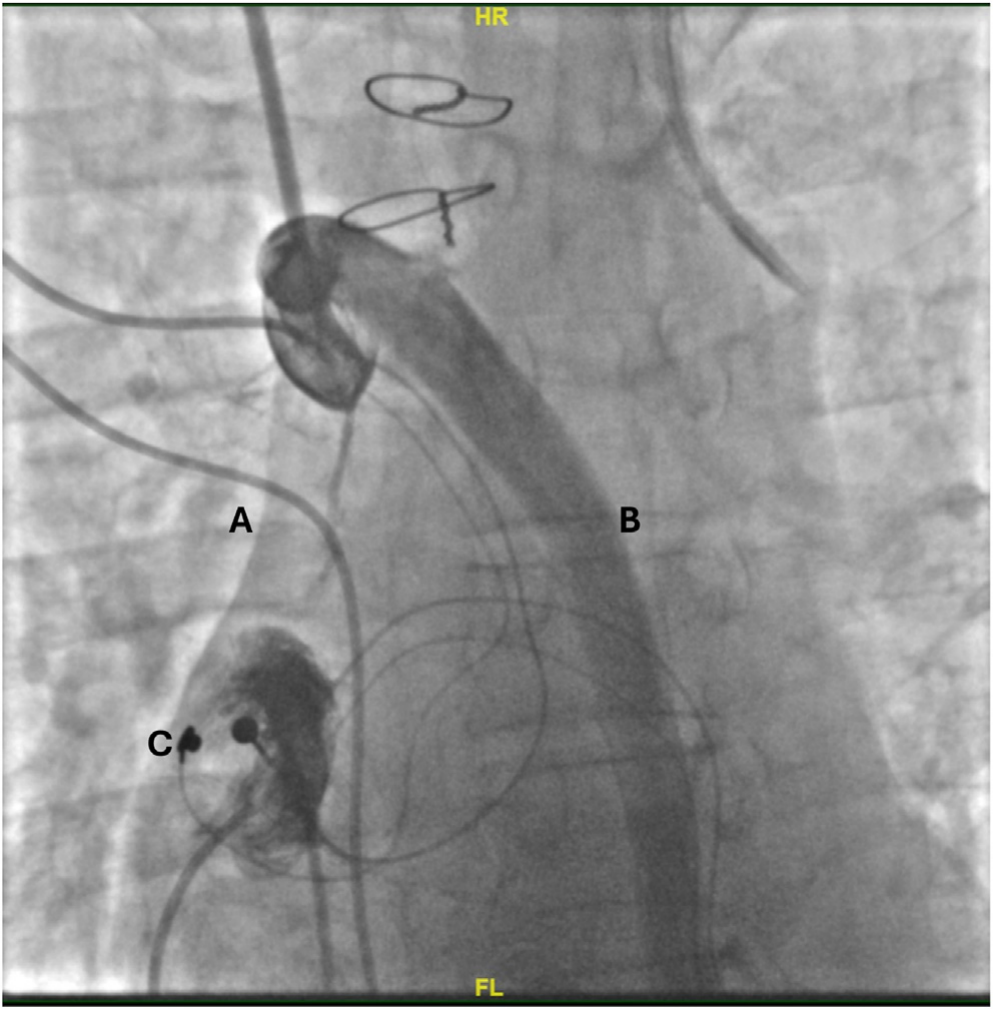
Dual venogram confirming total superior vena cava (SVC) occlusion. **A:** SVC; **B:** azygos vein; **C:** epicardial pacemaker leads.

**Figure 2:**
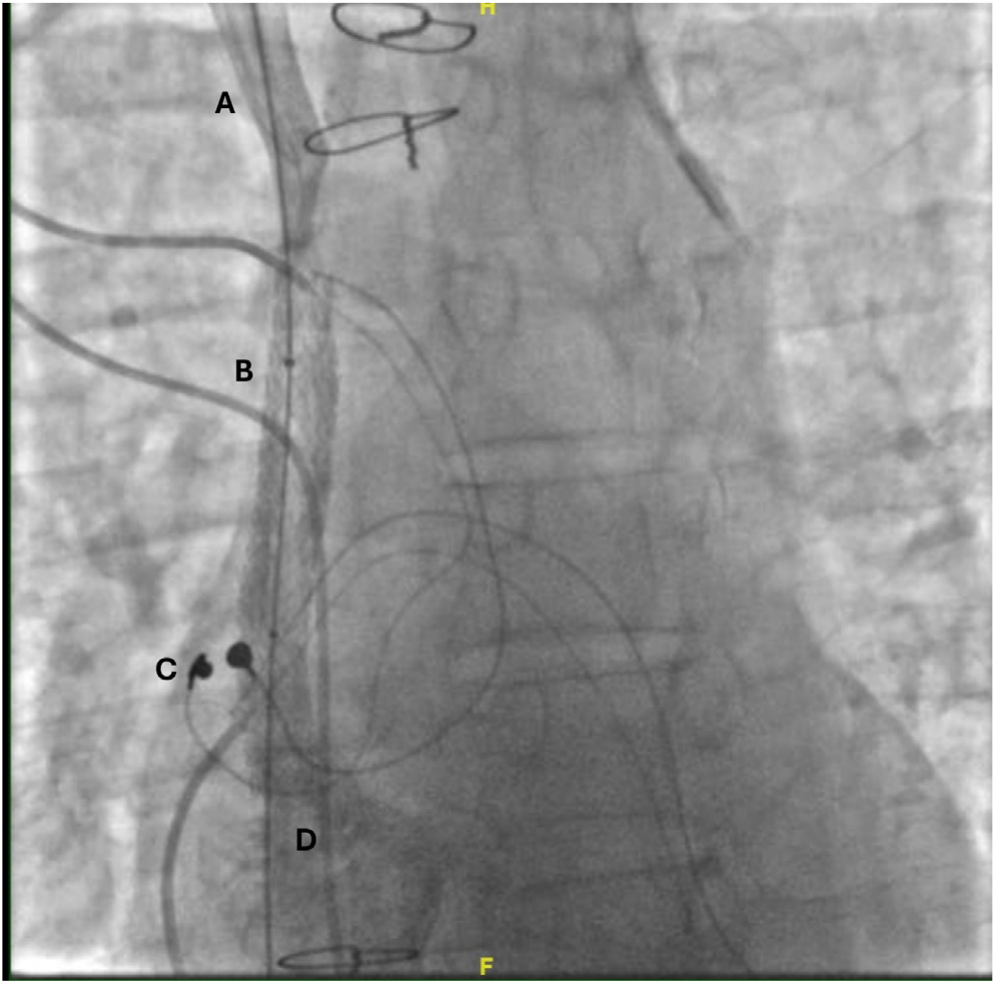
Post stent deployment. **A:** Brachiocephalic vein; **B:** deployed stent post creation of the de novo channel; **C:** epicardial pacing leads; **D:** contrast into the right atrium.

**Figure 3:**
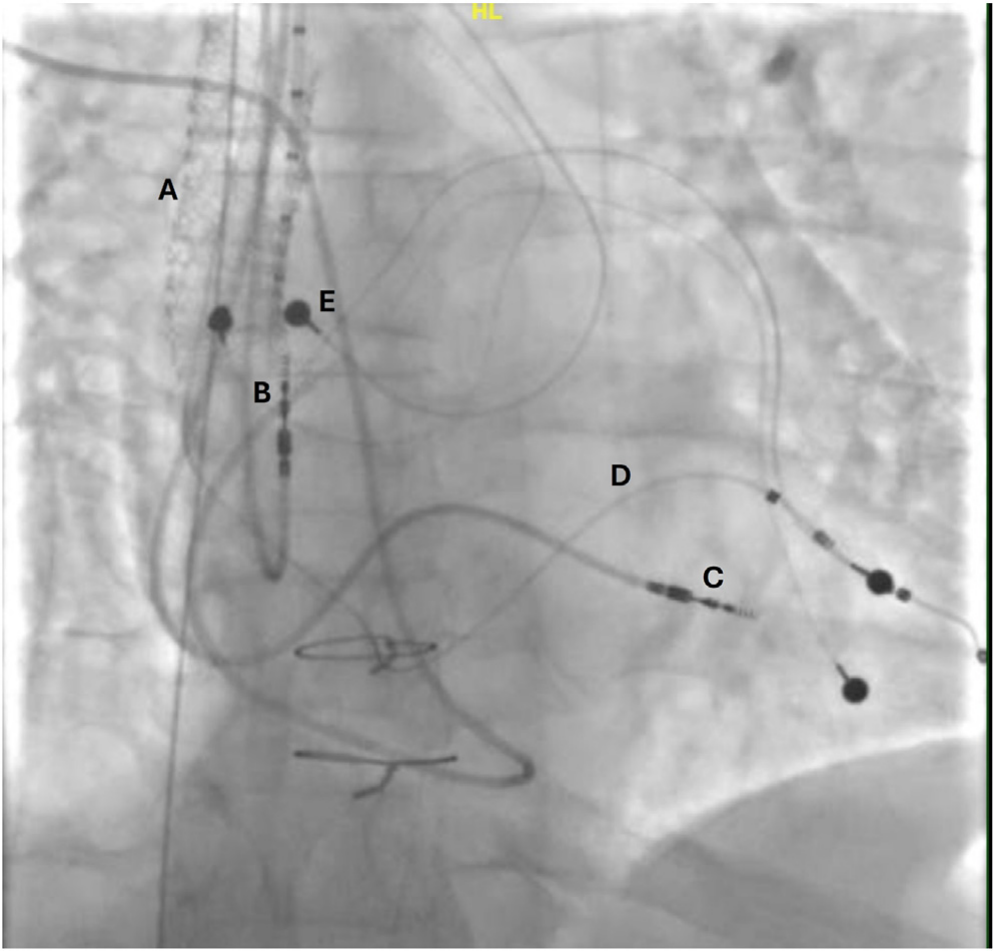
Cardiac resynchronization therapy pacemaker device insertion using the conduit. **A:** Optimized channel post insertion of second stent; **B:** atrial leads; **C:** right ventricular lead; **D:** left ventricular lead; **E:** epicardial pacing lead.

Preprocedure angiogram (Figure 1) and computed tomography venogram confirmed complete occlusion of SVC from origin to right atrium (RA). We then proceeded to create a new channel within the extravascular mediastinal space. Dual access was obtained via right internal jugular and femoral vein. An Amplatz gooseneck snare catheter (Medtronic, Minneapolis, MN) was advanced femorally to the RA. Superiorly, after accessing the mediastinum through the SVC at the level of occlusion, an Astato XS 20 wire (Asahi Intecc, Sato, Japan) was advanced with microcatheter support through an external Quick-Cross (Philips, Colorado Springs, CO) guide catheter toward the RA until access was achieved. This was then snared within the RA and brought through to externalize from the right femoral venous access. The channel was then dilated and upsized to an Amplatz Super Stiff Guidewire (Boston Scientific, Marlborough, MA) to allow insertion and dilation of a 79 mm ViaBahn (W.L Gore & Associates, Newark, DE) stent, leading to formation of the de novo conduit, with excellent angiographic result (Figure 2). Two weeks later the channel was further optimized using an 18 mm ATLAS Gold (Bard Peripheral Vascular, Tempe, AZ) balloon and 36 mm LD max (Medtronic, Minneapolis, MN) stent. The right-sided cardiac resynchronization therapy pacemaker insertion was then carried out through the de novo channel (Figure 3), and the old epicardial generator was explanted and leads capped and buried subpectorally. Subsequent follow-ups demonstrated good biventricular pacing with improved ventricular synchrony and resolution of symptoms.

## Discussion

Pacing-induced cardiomyopathy and venous stenosis and obstruction post cardiac devices are relatively common, with reported prevalence of 12%^11^ and 30%–50%,^4^ respectively. However despite its prevalence, an international consensus on approaching this issue is lacking. While various approaches have been proposed,^2^^,^^12^^,^^13^ there is little in terms of comparison, and decision is predominantly based on available expertise and operator comfort. Creation of new conduits has been extensively studied within the congenital heart disease population,^14^ but the idea of creating a conduit solely for the purpose of device upgrade is relatively novel. This case expands our understanding by demonstrating the possibility of approaching SVC occlusion by creation of a de novo channel from SVC to RA, for the purpose of device upgrade by using the chronic total occlusion technique and snare assistance. While we achieved excellent short-term results, the long-term outcome of our approach remains to be seen.

## Disclosures

No conflicts of interest to disclose.
